# Evaluation of changes in postnatal care using the "Parents' Postnatal Sense of Security" instrument and an assessment of the instrument's reliability and validity

**DOI:** 10.1186/1471-2393-9-35

**Published:** 2009-08-12

**Authors:** Linda J Kvist, Eva K Persson

**Affiliations:** 1 Department of Obstetrics & Gynaecology, Helsingborg Hospital, Helsingborg, Sweden; 2 Division of Nursing, Department of Health Sciences, Lund University, Lund, Sweden

## Abstract

**Background:**

A sense of security is important for experiences of parenthood in the early postpartum period. The objectives of this study were to evaluate two models of postnatal care using a questionnaire incorporating the Parents' Postpartum Sense of Security (*PPSS*) instrument and to test the validity of the *PPSS *instrument.

**Methods:**

Postal surveys were sent to 234 mothers who had experienced two different forms of postnatal care (study group and control group) and returned by 86.8%. These two groups of mothers were compared for total scores on the *PPSS *instrument. Demographic variables and mothers' opinions about care interventions were also compared and these variables were tested for correlations with the total *PPSS *score. A regression analysis was carried out to assess areas of midwifery care which might affect a sense of security. The internal consistency and concurrent validity of the instrument were tested for the total population.

**Results:**

there were no significant differences between the groups for scores on the *PPSS *instrument. A total of three variables predicted 26% of the variability on the *PPSS *scores for the study group and five variables predicted 37% of the variability in the control group. One variable was common to both: "*The midwives on the postnatal ward paid attention to the mother as an individual"*. There were significant correlations between the total *PPSS *scores and scores for postpartum talks and visits to the breastfeeding clinic. There was also a significant correlation between the single question: "*I felt secure during the first postpartum week*" and the total *PPSS *score. Tests for internal consistency and concurrent validity were satisfactory.

**Conclusion:**

The proposed new model of care neither improved nor impaired mothers' feelings of security the week following birth. Being seen as an individual by the midwife who provides postnatal care may be an important variable for mothers' sense of postnatal security. It is possible that postpartum talks may encourage the processing of childbirth experiences in a positive direction. Availability of breastfeeding support may also add to a sense of security postpartum. The *PPSS *instrument has shown acceptable reliability and validity.

## Background

A baby's interactions with its parents during the first weeks of life are an important pre-requisite for its continued wellbeing [1]. When parents begin to understand and react to the infant's basic needs they develop feelings of security about their new role as parents [1]. The ways in which provision of health care services during the early postnatal period may impact the new family is a subject which should be constantly under our surveillance. A recent Australian study has shown that, within existing models of care, women in the early postnatal period have fears and anxieties about their competence as new mothers and their ability to care for the new baby [2]. Studies show that mothers' and fathers' experiences during the childbirth period influence not only the child but also their own subsequent well-being [3-5]. Research has demonstrated effects of these experiences on parental relationships [3,4] and effects on the child have been suggested [6]. Earlier Swedish research into factors which influence choice and experiences of early discharge after childbirth has indicated the importance of a sense of security for the individual's experience of parenthood in the early postpartum period [7-9].

It was reported in 1995 that postnatal maternal morbidity was extensive and unrecognised [10] and since then numerous studies of maternal postnatal wellbeing have considered physical, psychological and psychosocial problems [11-15]. Lonstein [6] states that many women experience emotional disregulation and Skari et al. [16] showed in their Norwegian study that 37% of the mothers and 13.5% of the fathers reported psychological distress such as anxiety and depression, a few days after childbirth. In a Swedish study which measured women's satisfaction with their postnatal care at two months and one year, 26% of women were not satisfied with their postnatal care and amongst the risk factors for dissatisfaction was the presence of many physical symptoms [14]. The authors of the study discussed the possibility that although anxiety and depression seemed not to be associated with low satisfaction it was possible that these problems caused some amount of somatisation which then appeared as a risk factor for dissatisfaction.

According to some research [17] support, counselling, understanding and information, given to women by midwives in the postnatal period may provide benefits to psychological wellbeing although a review of the literature showed that the evidence for the use of postpartum talks was inconclusive [18]. Persson and Dykes [7] found that early postnatal security was dependent on parents' perceptions of the midwife's empowering behaviour, a sense of affinity within the family, a sense of autonomy and control and a sense of wellbeing which included manageable breastfeeding. Based on these results an instrument (*"Parents' Postnatal Sense of Security", PPSS*) is under development [19] which may be used to evaluate whether care offered enhances feelings of security during this sensitive period.

Although there are exceptions to the rule, maternity care in Sweden continues to a great extent to be fragmented, despite attempts to create services where midwives alternate between antenatal, birthing and postnatal care units. Continuity of care giver through the childbearing process is not a common feature of usual maternity care in Sweden. Midwives are the primary care providers for women at low risk of complications and these midwives are mostly community-based. It is these midwives who provide preparation for parenthood classes. In Sweden postnatal care has undergone changes, particularly regarding the length of stay, but also regarding the amount of own responsibility that new parents are expected to take [20]. Puerperal care of the mother and baby has in the last two decades been considerably reduced from the six weeks stipulated by the WHO in 1998 [21]. The length of hospital stay in Sweden for healthy primiparous women experiencing a normal delivery has decreased over a fifteen year period from approximately one week to 48 hours.

The organisation of postnatal care of families after discharge differs from hospital to hospital according to the preferences of the obstetrician-in-charge of the local obstetrics and gynaecology unit. Most hospitals require the mother and baby to stay at the hospital for observation for a minimum of six hours after the birth. Paediatricians' preferences determine the timing of examination of the newborn baby which also varies between hospitals. At some hospitals the baby is examined at six hours of age, before discharge and at others the family may leave the hospital without examination of the baby and return for examination when the baby is approximately 12 hours old. Some hospitals offer home visits by a midwife from the hospital where the birth took place, some offer telephone follow-up and others require the parents themselves to make contact with the hospital maternity services if needed. During the first days, advice and information is given regarding breastfeeding, care of the newborn infant and the health of both mother and infant. After this period, a short written summary of the birth and early postnatal period is sent to the district nurse who takes over responsibility for care of the baby and the breastfeeding dyad. Within 10 days of discharge the district nurse carries out a home visit.

In the Australian study which showed that women in the early postnatal period have anxieties and fears regarding their parenting role, the authors suggested that care providers should be sensitive to the needs of individuals when planning postnatal services [2]. In Sweden, shortened length of puerperal care has been implemented with little recognition or concern for what these changes might entail for the health and welfare of the new family. It is of great importance that changes in postnatal care are evaluated both in relation to safety and parents' experiences of security.

At a hospital in southern Sweden a development project has been carried out which aimed to establish a new model of care by making the antenatal midwife the first line carer for mothers discharged from hospital but not yet in the care of the district nurse. Midwives at the antenatal clinics work to a case load and the rate of continuity of care giver before the birth is high in the whole district (unpublished data). The project aimed to increase continuity of care giver *after *the birth and to make maximum use of the relationship to and knowledge of the family which the antenatal midwife builds up during the months of pregnancy. Regional guidelines for care in pregnancy state that the appropriate number of midwife visits for women with a low-risk pregnancy is approximately eight.

At the hospital in question there are two wards for care after birth; one family-oriented ward and one traditional postnatal ward which cares for mothers with birth complications. The family-oriented ward also has a breastfeeding clinic, available for booked visits all days of the year and which is staffed by the midwives who work on the ward. No home visits are carried out after discharge from this hospital. Women are given telephone numbers where they can reach a hospital midwife 24 hours a day. Midwives occasionally make a spontaneous telephone call to women for whom they feel some kind of concern. Families return to the family-oriented ward for follow-up visits after discharge. The midwives alternate between the family ward and the traditional postnatal ward, which means that it is in no way certain that the woman will recognise the midwife whom she meets for the follow-up visit. Earlier research carried out at this hospital regarding fear of childbirth [22,23] has increased awareness amongst the midwives about the value of postpartum talks. However, short hospital stays can make it difficult for the midwife who attended the woman at the birth to talk to the mother before she leaves the hospital.

It is important that new forms of care are evaluated to ensure that services are not weakened for new families. Rates of neonatal re-admissions were monitored in the project but no scientific evaluation of the effect the new model of care has on parents' postpartum experiences has previously been carried out.

The objectives of this study were to evaluate two models of postnatal care using a questionnaire incorporating the *PPSS *instrument, and to test the reliability and validity of the instrument.

## Methods

In this study, mothers who experienced the new model of care are referred to as the study group and mothers who did not experience the new model of care are the control group. The hypothesis tested was that care in the early postnatal period given by the midwife who cared for the woman during pregnancy (study group) and who is therefore known to her, will result in a greater sense of security in the mother than care given by the hospital midwife, who is, for the mother, likely to be an unknown person (control group). The *PPSS *instrument was incorporated into a questionnaire to allow for analysis of variables which may affect a sense of security, independent of the two models of care. This questionnaire has been used and tested in earlier research for the development of the *PPSS *instrument [24].

### The two different models of care in the project

**Figure 1:**
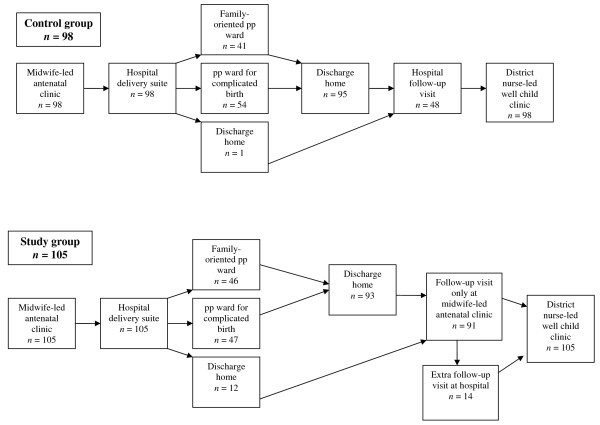
**The two models of care compared in the study**. *n *= the number of individuals completing the box-event.

Figure 1 shows the two different models of care that were evaluated in the study. There were two major differences in the new model of care; the locality of the follow-up visit and that the midwife whom the woman met at the follow-up visit was her antenatal midwife.

### Participants and procedure

Two antenatal clinics of similar size within the same uptake area participated in the study; one where postnatal mothers were cared for by antenatal midwives and one where postnatal care was given by hospital midwives. The two clinics were part of the same organisation and located in the same town. When the study started all of the women were already booked at the respective clinics and therefore did not make a choice in the model of postnatal care that they would receive. Midwives work to a case load and therefore staffing levels were the same at the clinics and provision of care did not differ in any obvious way apart from the postnatal follow-up.

All women with expected date of delivery between January and April 2008 were invited to join the study which would entail completing a questionnaire. Information was given to them by their midwife at the antenatal visit nearest 36 gestational weeks, explaining that they would be asked to answer a questionnaire about their experiences during the first postnatal week. The questionnaire would be sent to them during the second postnatal week. They were informed that none of the midwives responsible for their care would be aware of answers given and that refusal to join the study would in no way affect their care. They were free to change their mind about participation at any time. Mothers who had a stillbirth or gave birth to a baby who was not well were excluded as were mothers who did not have sufficient language skills to complete the questionnaire. All of the midwives working at the clinic were involved in the study and at the end of each month they were asked to provide lists of potential respondents who had been excluded and the reasons for the exclusions.

Number-coded questionnaires were sent by post to all participants together with an envelope with pre-paid postage which was addressed to the first author. A reminder and a new questionnaire were sent if the questionnaire had not been returned within two weeks.

### The questionnaire

#### Demographic variables and questions regarding care received

Demographic and background questions regarding items which theoretically may affect sense of security included age, education level, parity, country of birth, other language than Swedish spoken at home, unemployment, baby born post-maturely (> 42 weeks), whether parenthood classes were attended, whether the partner was present at the birth, the mode of birth and whether a postpartum talk was carried out by the midwife assisting at the birth.

Earlier research has indicated variables that may have some effect on mothers' experiences of a sense of security the week following birth [24]. These variables formed a further fifteen items that referred to midwifery care and concerned feelings of being given attention by the midwife, experiences of participation in care both for self and for the partner, physical and psychological health and feelings of security before the pregnancy, being seen as an individual and being encouraged by the midwifery staff, having a positive expectation and a positive experience of the delivery, being kept informed, experiences of participation in care for self and partner and participation in decision-making. All these items were answered on 4-point Likert-type scales where answers were: "I agree completely", "I agree quite a lot", "I agree to a little degree" and "I do not agree at all".

Questions were also posed regarding the form of hospital care given (family- oriented, care for complications or home directly from delivery suite), time for hospital discharge and whether discharge time was mothers own choice. Mothers were asked about antenatal preparation and postnatal follow-up by ranking the usefulness of parenthood classes, postpartum talks, telephone follow-up after the birth and contacts with the breastfeeding clinic. Postpartum talks were determined as "positive", "negative" or "indifferent". Parenthood classes, telephone follow-up and the breastfeeding clinic were ranked on 4-point Likert-type scales of the same type as above. The mothers were asked to gauge their general feelings of security in one global question: "I felt secure the first week after the birth", to which they could answer: "Not at all", "To some degree", "Quite a lot" or "Completely".

### The PPSS instrument

The two models of postpartum care were evaluated using the *PPSS *instrument which is under development [19]. The instrument is being developed for use with both parents but for this study only the mothers' sense of security was measured. The maternal version of the *PPSS *instrument contains 18 items concerning the week following delivery which include questions regarding staff attitudes, attention, information and advice given, physical and psychological health, feelings of participation in care, feelings of affinity within the family, availability of care providers, experiences of breastfeeding, and feelings of security. The items are in the form of statements to which the mother is asked if she agrees: completely, quite a lot, to a little degree or not at all. Scores on the instrument may range from 0 to 72 points. A preliminary test of the instrument [19] showed a mean of 56.3 (SD 9.6, range 18 – 72). A power calculation (α = 0.05, β = 0.2) based on these figures and hypothesising a 10% increase for mothers in the study group for mean scores on the *PPSS *instrument (from 56.3 to 62.0) showed a requirement of 89 participants in each group.

Not all responders answered all the 18 items on the *PPSS *instrument and therefore a minimum of 16 answered items was required for inclusion in the analysis. The multiple imputation method was used for questionnaires with missing answers for one or two of the 18 items, in order to allow them to be included in the analysis. The requirement was fulfilled by 100 mothers in the study group and 94 in the control group. Thus the study was adequately powered for comparison of total *PPSS *scores. Fourteen of the mothers in the study group visited the hospital for an extra follow-up visit because the antenatal clinic was closed due to holidays. These mothers also had a postnatal visit with their antenatal midwife when the clinic re-opened.

### Ethical approval

Ethical approval was requested from the Committee for Medical Research Ethics, Lunds University Hospital, Sweden. The committee considered that the research was not of a character that might incur any substantial risks for the participants (protocol number 2007/11).

### Analysis

The data were processed using SPSS version 15.0. The study and control groups were compared for demographic variables and for variables that might conceivably effect mothers' sense of security (attendance at parenthood classes, accompanied by partner at antenatal visits, partner present at birth, normal birth, premature birth and no postpartum talk), using the student's t-test for continuous variables and the Chi square test for categorical variables. Fisher's exact test was used when very small numbers were expected in the analysis of proportions. Results for continuous variables are presented with means and standard deviations and categorical variables with frequencies and percentages. Total scores for the *PPSS *were compared between the groups using student's t-test. Scores for the usefulness of the breastfeeding clinic, parenthood classes, postpartum talks, telephone follow-ups and also quality of contacts with the breastfeeding clinic were dichotomised; "I agree completely" and "I agree quite a lot" were given one value and, "I agree to a little degree" and "I do not agree at all" were given another value. The two groups were compared for differences using the Chi square test (Fisher's exact test). These variables were also tested for correlations with the total *PPSS *score, using Spearman's correlation coefficient (*rho*).

Possible determinants for mothers' postnatal sense of security were examined using regression analysis [25]. These possible determinants were the 15 items about midwifery care. Answers to the items were dichotomised; "I agree completely" and "I agree quite a lot" became one value and answers "I agree to a little degree" and "I do not agree at all" became another value. A multiple, linear, stepwise regression analysis of all the 15 items which had the potential to affect the *PPSS *score was carried out and the risk for collinearity was tested using VIF (Variance Inflation Factor) and Tolerance [25].

Cronbach's alpha was used to assess reliability of the instrument by testing the items on the *PPSS *instrument for internal consistency. Concurrent validity [26] of the instrument was assessed by calculating Spearman's correlation coefficient between the one global question *"I felt secure the first week following delivery" *and the total scores for the 18-items on the *PPSS *instrument. All tests were two-tailed and a p-value of 0.05 or smaller was used to indicate statistical significance (*p*-value).

## Results

During the study period 294 mothers were eligible for participation. Fourteen mothers declined the invitation, the midwives forgot to invite six mothers, 35 did not understand the Swedish language sufficiently well to participate and five had an infant whose state of health was uncertain at the time the questionnaire should be sent, giving an external exclusion rate of 20%. A total of 234 questionnaires were sent to mothers who agreed to participate, 124 to mothers in the study group and 110 mothers in the control group. The questionnaire was returned by 84.7% (*n *= 105) in the study group and 89.1% (*n *= 98) in the control group, giving an overall response rate of 86.8%.

### Comparisons of background variables between the study group and the control group

Table 1 shows comparisons of background variables for the two groups. There were significantly more mothers (*p *< 0.01) in the study group who had a college or university education and the mean age for this group was significantly higher than the control group (*p *< 0.01). There were no differences between study and control groups for numbers cared for on the two hospital wards before discharge but there was a difference in the number of mothers who chose discharge direct from the delivery suite (6 hours postpartum); 12 in the study group and 1 in the control group (*p *< 0.01). Almost all of the mothers, 85% (*n *= 89) in the study group and 91% (*n *= 89) in the control group felt that they themselves had chosen the timing of their discharge.

**Table 1:** Comparison of background variables between the study and control groups

	**Study group** *n *= 105	**Control group** *n *= 98	***p-value***
**Variables**			
Age in years, mean (SD)	32 (± 5.4)	29 (± 5.6)	< 0.01
College or university education	64 (60.9%)	38 (38.7%)	< 0.01
			
Primiparous	46 (43.8%)	48 (48.9%)	0.550
Born outside Sweden	17 (16.2%)	17 (17.3%)	0.974
Other language spoken at home	12 (11.4%)	14 (14.2%)	0.690
			
Unemployed	11 (10.5%)	17 (17.3%)	0.225
Attended parenthood classes	56 (53.3%)	43 (43.8%)	0.228
			
Partner accompanied to antenatal visits	81 (77.1%)	79 (80.6%)	0.665
Partner present at the birth	98 (93.3%)	95 (96.9%)	0.389
Normal vaginal birth	85 (80.9%)	79 (80.6%)	1.000
Baby born post-maturely	6 (5.7%)	5 (5.1%)	1.000
No postpartum talk with a midwife	37 (35.2%)	36 (36.7%)	0.940

There were between 1 and 3 non-responders in the study group for five questions and between 1 and 2 in the control group for four questions

Mean age was tested with t-test, all other variables tested with Chi^2^

Eleven percent (*n *= 11) in the study group and 12% (*n *= 12) in the control group answered that they received a telephone follow-up. A further 28% (*n *= 29) in the study group and 24% (*n *= 23) in the control group would have liked to receive telephone follow-up. Questioning about postpartum talks showed that 64% (*n *= 67) in the study group and 60% (*n *= 59) in the control group had had a postpartum talk with a midwife, Chi-square 0.30; *p *= 0.67 and that a further 27% (*n *= 28) in the study group and 25% (*n *= 24) in the control group would have liked to have had a talk. None of the mothers answered that the talk was a negative experience. Of the mothers in the study group 77% (*n *= 77) and 82% (*n *= 70) in the control group answered that the breastfeeding clinic was a useful or very useful service. In the control group 20% mothers (*n *= 21) in the study group and 19% (*n *= 19) had been in contact with the breastfeeding clinic and they ranked these contacts as positive. Significantly more mothers in the study group perceived parenthood classes as not useful (*n = *13) than in the control group (*n *= 3), Chi-square (Fishers) = 4.32; *p *= 0.03. There were no differences between the groups for percentage of partners who slept over at the postnatal wards; in the study group 82.7% (*n *= 81) and in the control group 85.6% (*n *= 83) of fathers had this opportunity to remain with the family.

### Experiences of pregnancy and birth in the study group and the control group

Comparisons between the groups for the six items pertaining to mothers' experiences during pregnancy showed a significant difference for one item. Mothers in the control group (*n *= 98) felt that they had participated in their care to a higher degree than the study group (*n *= 103) Chi-square = 6.68; *p *= 0.006. There were no significant differences between the groups for any of the nine items which referred to care during birth.

### Group comparisons of mothers' postnatal sense of security

#### Experiences the week following birth

No statistically significant difference was found between the two groups for mothers' postnatal sense of security measured by the total score on the *PPSS *instrument; mean score for the study group was 55.1 (± 6.0) and mean score for the control group was 55.5 (± 6.0).

### Correlations between total PPSS scores, antenatal preparation and postpartum follow-up in the total study population

Irrespective of group, there were significant correlations between the total *PPSS *scores and women's positive views on the usefulness of postpartum talks, *rho *= -0.18 (*p *= 0.04). There was also a significant correlation between total *PPSS *scores and women's positive views on the usefulness of the breastfeeding clinic, *rho *= 0.24 (*p *= 0.004). There were no significant correlations between the total score and views on preparation for parenthood classes or telephone follow-ups. Women who had a postpartum talk (*n *= 126) had a mean score for the total *PPSS *of 56.3 (± 5.8) and women who did not have a postpartum talk (*n *= 73) had a mean score of 53.7 (± 6.2). The difference was statistically significantly; *t *= 2.99, *p *= 0.003.

### Potential determinants for postnatal sense of security in the study and control groups

Tables 2 and 3 show results of the multiple linear analyses of the 15 dichotomised items pertaining to experiences of midwifery care.

**Table 2:** Regression model for determinants for *PPSS *scores in the study group and collinearity statistics.

**Determinants for *PPSS***	**Unstandardized regression coefficient**	**95% CI**	***P*-value**	**Collinearity statistics**	
				**Tolerance**	**VIF**
*I felt that the midwives on the postnatal ward* *paid attention to me as an individual*	6.04	3.09 – 9.05	< 0.001	0.95	1.06
*I felt that my partner participated in my care* *during the birth*	3.85	1.80 – 6.72	0.20	0.98	1.02
*I felt that I participated in decision making* *during the birth*	3.20	0.03 – 6.34	0.05	0.96	1.05

Adjusted R square = 0.26

**Table 3:** Regression model for determinants for *PPSS *scores in the control group and collinearity statistics.

**Determinants for *PPSS***	**Unstandardized regression coefficient**	**95% CI**	***P*-value**	**Collinearity statistics**	
				**Tolerance**	**VIF**
*I felt that the midwives on the postnatal ward* *paid attention to me as an individual*	6.38	3.09 – 9.05	< 0.001	0.95	1.05
*I felt that my psychological health was good* *during pregnancy*	3.97	1.80 – 6.72	0.04	0.96	1.04
*I had positive expectations of the birth*	2.84	0.65 – 5.03	0.01	0.99	1.01
*I felt that my partner participated during my* *pregnancy*	4.13	1.28 – 6.98	0.01	0.92	1.09
*I felt that I participated in my care during* *pregnancy*	-3.96	-7.77 to -0.16	0.04	0.94	1.06

Adjusted R square = 0.37

A model containing three of the fifteen items could explain approximately 26% of the variation in *PPSS *scores in the study group and for the control group a model containing five items could explain approximately 37% of variation on the *PPSS *scores. One item was common to both the study and control groups, this was: *"I felt that the midwives on the postnatal ward paid attention to me as an individual"*. Tables 2 and 3 also show the results of the VIF and Tolerance tests to assess the amount of inter-relatedness of the items.

### Reliability and validity of the PPSS

**Figure 2:**
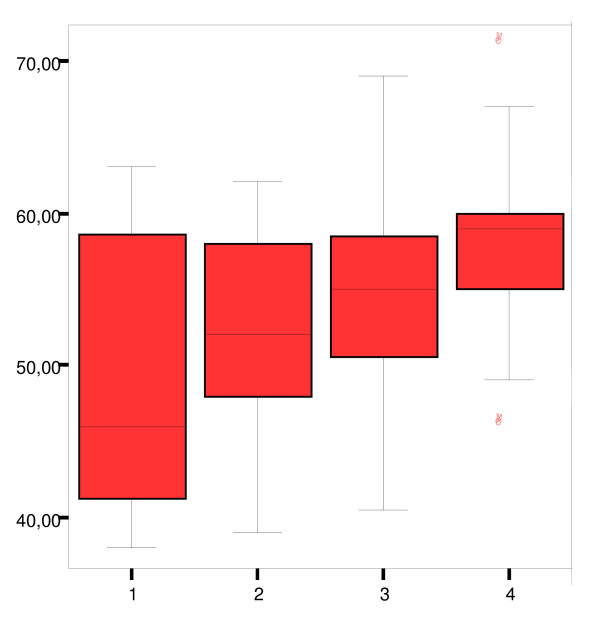
**Relationship between mothers' scores for *PPSS *and the global question on postpartum security in the total population**. The x-axis denotes the scores (1–4) for the global question" *I felt secure during the first postnatal week"*, and the y-axis denotes the total scores (0–72) for the *PPSS *instrument. As scores increase for the global question, results of scores on the *PPSS *become increasingly homogenous and the inter-quartile range decreases.

The Cronbach's alpha coefficient for internal consistency in the *PPSS *instrument was 0.85. Concurrent validity between the global question: "*I felt secure during the first postnatal week*"and the total *PPSS *score for all study participants showed a Spearman's correlation coefficient of 0.36 (*p *< 0.01). Figure 2 shows a box-plot of the relationship between the global question and the *PPSS *scores for the whole study population. It can be seen that the higher the score (1–4) for the global question, the more homogenous the scores for the *PPSS *instrument. The median score for *PPSS *gradually increases and the inter-quartile range decreases with increments in scores for the global question.

## Discussion

Results showed that the changes in the model of care for women during the first postpartum week which the project entailed neither increased nor decreased mothers' sense of security as measured by the *PPSS *instrument. One interpretation is, that for the population examined in this study, knowing the identity of the midwife who gave care during the postnatal period did not increase a sense of security. Some earlier research regarding continuity of care giver in maternity services has shown similar results [27,28]. The fact that significantly more mothers in the study group chose discharge directly from the delivery suite, 6 hours after birth, may on the other hand have been an indication that they felt secure in the knowledge that they would be followed-up by their antenatal midwife. However, the figures are small and should therefore be interpreted with caution. The differences in the two models of care were relatively small, the main differences being the person conducting the follow-up and its location. Therefore a 10% increase in a sense of security may have been a rather high figure to expect. This figure was hypothetical and was used in order to enable a calculation of power for the study.

The attempt to measure care which was given within a very specific time frame meant that the *PPSS *instrument was administered during a period when parents may be feeling relieved that mother and baby have survived the ordeal of birth. This "halo-effect" may have had some influence on the participants' responses and the results should be considered with this in mind. However, both groups answered the questionnaire during the same period of time. It was considered that it was necessary to send the questionnaire as close to the period of care as possible in order to minimise the effect of blurring of care episodes. Since the model of care neither improved nor worsened women's postpartum sense of security, the decision to adopt the model or not may instead be based on other considerations, for example, job satisfaction for the midwives and which model is most economically justifiable.

It appears that the differences between the two groups for education levels and age may indicate a difference in socio-economic groupings. It is uncertain to what extent the fact that mothers in the study group had significantly higher levels of education and were significantly older than the control group affected the results of the study. The fact that mothers in the control group experienced preparation for parenthood classes as significantly more useful than the study group could possibly have negated any effect that age and education might otherwise have had on the total *PPSS *score. This difference is, on the other hand, compounded by a significant difference in the feelings of participation in antenatal care experienced by mothers in the control group and may be due to a differing culture at the two antenatal clinics. It would be of interest to further investigate the reasons for differences in experiences of parenthood classes and participation in antenatal care as part of quality development.

At the unit under investigation, timing of discharge is by parents' personal preference, on the condition that all is well with mother and baby. Therefore the result that almost all felt the choice of discharge time to be their own was not surprising but never the less gratifying since it has been shown that being sent home involuntarily can lead to dissatisfaction with care [29]. Mothers were not asked in this study whether they considered the length of their hospital stay to be appropriate and this question could have been pertinent since there may be societal pressure on new mothers to prove their "competence" by choosing discharge early after birth. Those who return home quickly after birth are in common Swedish parlance often referred to as "capable". Home visits by the midwife are not part of the care plan at this unit and it is maybe for this reason that a little more than a quarter of the respondents answered that they would have liked to have had a follow-up telephone call.

Women who had had a postpartum talk in the present study scored significantly higher on the *PPSS *than those who did not have a postpartum talk which suggests that these talks may be a means of starting to process the experiences of childbirth. Approximately 62% of the mothers had a talk with a midwife and a further 26% would have liked to have had a talk. These results are very similar to a recent Swedish study [30] showed that 56% of women had been through a postpartum "consultation" and that approximately 25% would have liked to have had a consultation.

Being seen as an individual by the midwives on the postnatal ward was an important variable for a postpartum sense of security for the whole study population. The importance of the principal of individualised care has been established in a guideline for care in the postpartum period published in the UK by the National Collaborating Centre for Primary Care [31]. Behaviour of this kind can be expressed as empowering behaviour; the midwife sees the individual and her individual needs and tailors care to meet these needs. Researchers have discussed the advantages of this kind of behaviour earlier [7,32,33]. It has been noted that feelings of disempowerment and devaluation are among the feelings which underpin most reports of negative experiences in health care [34]. Discussions of empowering behaviour and even role-playing could be incorporated into midwifery education.

It has earlier been hypothesised that mothers' experiences during pregnancy, birth and postnatal care may have some effect on their sense of security the week following birth [24] and the regression analysis in the present study showed that between 26% and 37% of variations in *PPSS *scores were determined by items pertaining to maternity care. However, the number of items entered into the regression model was rather high considering the size of the study population and therefore these results will require further investigation. Although there is no formal "cut-off" value for VIF, values above 10 are usually taken to suggest strong inter-relatedness of the variables and in weaker models, values of above 2.5 may give cause for concern [35]. Therefore the results of the tests for collinearity suggest that the models may be reasonably robust. The fact that items in the two regression models were not identical may be a result of the differences in education levels and age between the two groups. This suggests that the instrument may have sensitivity to different study populations which may make it useful in varying research settings.

It was shown in an earlier study [7] that manageable breastfeeding was an integral part of feelings of security in the postpartum period. It was also seen in the present study that a positive view of the usefulness of a breastfeeding clinic was associated with a postnatal sense of security. The very availability of a service for help with breastfeeding questions and problems may help to reduce anxiety about the breastfeeding situation and thereby lend subliminal support to new mothers.

There has been a recent surge in the amount of postnatal care research published in peer-reviewed journals. If postnatal care is to rid itself of the epithet of the "Cinderella service" of maternity care this trend needs to continue. Research into differing aspects of this important period of adjustment for new families must be initiated by midwives. It is at present not possible to say which levels of scores on the *PPSS *may denote security or insecurity since the instrument is still under development and will require further testing. However, both the results of the Cronbach's alpha and the Spearman's correlation test (0.36, *p *< 0.01) suggest that the reliability and validity of the *PPSS *instrument may be acceptable since the *p-*value of < 0.01 denotes that there is a 1% risk that the results have occurred by chance. Reasons for feelings of security after childbirth are most certainly very diverse and culturally dependent. Further development of the *PPSS *instrument is underway and it will be important to test the instrument in varying cultural settings.

## Conclusion

The proposed new model of care neither improved nor impaired mothers' feelings of security the week following birth. Being seen as an individual by the midwife who provides postnatal care may be one of the most important variables for mothers' sense of postnatal security. It is possible that postpartum talks may encourage the processing of childbirth experiences in a positive direction. Availability of breastfeeding support may also add to a sense of security postpartum. The *PPSS *instrument has shown acceptable reliability and validity.

## Competing interests

The authors declare that they have no competing interests.

## Authors' contributions

LJK and EKP designed the study and LJK carried out data collection. Statistic analyses were carried out by LJK and EKP. LJK drafted the manuscript and changes were made according to suggestions from EKP. Both authors read and approved the final manuscript.

## Pre-publication history

The pre-publication history for this paper can be accessed here:


